# Replantation of amputated fingertips: reconstruction of circulation via proximal subungual arch artery or nail bed vein anastomosis

**DOI:** 10.3389/fsurg.2026.1851574

**Published:** 2026-06-29

**Authors:** Heyun Cheng, Heng Xie, Shuang Liu, Shuai Dong, Jihui Ju, Qiang Zhao, Benyuan Wang, Quanwei Guo, Xiaoyu Yu, Kai Wang

**Affiliations:** Department of Hand Surgery, Suzhou Ruihua Orthopedic Hospital, Suzhou, China

**Keywords:** aesthetic reconstruction, blood supply, digit replantation, fingernail, microsurgery

## Abstract

**Background:**

If no palmar digital artery or subcutaneous vein is available for anastomosis in the amputated distal fingertip tissue, conventional replantation methods cannot effectively restore blood circulation. This study introduces a surgical approach and evaluates its outcomes for replantation of distal fingertip amputated tissue when the palmar digital artery is unavailable for anastomosis. In such cases, the proximal subungual arch artery is anastomosed as an alternative to the palmar digital artery to restore arterial supply, while standard anastomosis of the dorsal subcutaneous vein is performed to restore venous drainage. When the subcutaneous vein is unavailable, the proximal nail bed vein is anastomosed as an alternative to the subcutaneous vein to restore venous drainage, while standard anastomosis of the palmar digital artery is performed to restore arterial supply. The aim is to improve the replantation rate, survival rate, and survival quality of amputated distal fingertip tissue.

**Methods:**

From January 2019 to December 2025, our institution performed replantation surgery on three patients with three digits of distal fingertip amputated tissue lacking a palmar digital artery for anastomosis. In these cases, the proximal subungual arch artery was anastomosed as an alternative to the palmar digital artery to restore arterial supply, while standard anastomosis of the dorsal subcutaneous vein was performed to restore venous drainage. Additionally, replantation surgery was performed on nine patients with nine digits of distal fingertip amputated tissue lacking a subcutaneous vein for anastomosis. In these cases, the proximal nail bed vein was anastomosed as an alternative to the subcutaneous vein to restore venous drainage, while standard anastomosis of the palmar digital artery was performed to restore arterial supply. Intraoperative measurements included the diameter of the proximal subungual arch artery or proximal nail bed vein used as an alternative vessel, and the duration of finger ischemia as well as the operative time were recorded. Postoperatively, the survival of the replanted tissue was observed, and complications such as vascular crisis, wound infection, nonunion of the fracture, and tissue necrosis were assessed. At the final follow-up, the function of the affected finger was evaluated using the Fingertip Injuries Outcome Score (FIOS).

**Results:**

In the 3 patients (3 digits) who underwent anastomosis of the proximal subungual arch artery as an alternative to the palmar digital artery, the arterial diameters were 0.2 mm, 0.3 mm, and 0.3 mm (mean: 0.27 mm). In the 9 patients (9 digits) who underwent anastomosis of the proximal nail bed vein as an alternative to the subcutaneous vein, the venous diameters ranged from 0.6 mm to 1.0 mm (mean: 0.72 mm). The mean warm ischemia time for the 12 amputated digits was 4.75 h (range: 3–11 h). The mean operative time for the 12 replantations was 1.97 h (range: 0.5–2.83 h). All 12 replanted digits survived, with no cases of vascular crisis, wound infection, fracture nonunion, or tissue necrosis. At a mean follow-up of 12.17 months (range: 3–43 months).The function scores of the affected fingers ranged from 11 to 15, with an average of 12.5. The results were rated as excellent in 6 cases and good in 6 cases.

**Conclusions:**

The proximal subungual arch artery and nail bed vein can be anastomosed under microscopic visualization. Anastomosis of the proximal subungual arch artery can replace the palmar digital artery to restore arterial supply to the distal amputated tissue block, while anastomosis of the proximal nail bed vein can replace the subcutaneous vein to restore venous drainage of the distal amputated tissue block. For patients with distal fingertip amputation injuries lacking a palmar digital artery or subcutaneous vein available for anastomosis, replantation can be performed by intraoperatively anastomosing the proximal subungual arch artery as an alternative to the palmar digital artery or the proximal nail bed vein as an alternative to the subcutaneous vein to restore arterial supply or venous drainage. This approach effectively improves the replantation rate, survival rate, and survival quality of the replanted tissue, and is worthy of widespread clinical application.

## Background

With the increasing mechanization of production, the incidence of finger amputation injuries has continued to rise (1). In the early stages, due to poor vascular anastomosis techniques and low success rates of replantation, amputation was often the standard treatment for finger amputation injuries, which severely compromised hand function and aesthetics (2, 3). However, with the rapid development of microsurgical techniques and increasing patient demands for both hand function and appearance, replantation of distal fingertip amputations has become a viable option to preserve most of the joint function of the affected digit. Successful replantation can maximize the restoration of finger function and aesthetics. Yano et al. considered replantation as the first-line treatment for distal fingertip amputation injuries (4).

The reconstruction of blood circulation is the most critical factor for the survival of replanted distal fingertip tissue (5, 6). Given the wide variety of distal fingertip amputation patterns, it is often found that no palmar digital artery or subcutaneous vein is available for anastomosis. Under such circumstances, conventional replantation techniques cannot effectively reestablish blood circulation, making replantation unfeasible.

This study aims to describe our approach for distal fingertip amputated tissue when the palmar digital artery is unavailable for anastomosis. In such cases, the proximal subungual arch artery is anastomosed as an alternative to the palmar digital artery to restore arterial supply. When the subcutaneous vein is unavailable for anastomosis, the proximal nail bed vein is anastomosed as an alternative to the subcutaneous vein to restore venous drainage. This provides a novel method for revascularization, improving the replantation rate, survival rate, and survival quality of distal fingertip amputated tissue.

## Patients and methods

### General data

A total of 12 patients with 12 digits were included in this study, comprising 8 males and 4 females, with a mean age of 42.83 years (range: 27–61 years). The right hand was involved in 6 patients (6 digits) and the left hand in 6 patients (6 digits). The injured digits included the thumb (7 digits), index finger (2 digits), middle finger (1 digit), and ring finger (2 digits). The causes of injury were cutting injury (6 cases), crush injury (4 cases), and avulsion/twist injury (2 cases). According to the amputation types, there were 3 cases of Ishikawa zone IV, 6 cases of Ishikawa zone III, 1 case of Ishikawa zone II, and 2 cases of dorsal fingertip tissue amputation. Among these, 3 cases had no available palmar digital artery for anastomosis, and 9 cases had no available subcutaneous vein for anastomosis (Table 1).

**Table 1:** Patient demographics, method of blood circulation reconstruction, and outcomes.

No.	Digit	Amputation type	Artery reconstruction	Vein reconstruction	Alternative vessel used	Artery diameter (mm)	Vein diameter (mm)	Follow-up (months)	FIOS score
1	Left thumb	Ishikawa Zone IV	Palmar digital artery	Proximal nail bed vein	Vein	Not measured	0.8	12	11
2	Right thumb	Ishikawa Zone IV	Palmar digital artery	Proximal nail bed vein	Vein	Not measured	1	17	11
3	Right ring finger	Ishikawa Zone III	Palmar digital artery	Proximal nail bed vein	Vein	Not measured	0.8	3	15
4	Left thumb	Ishikawa Zone IV	Palmar digital artery	Proximal nail bed vein	Vein	Not measured	1	20	11
5	Left thumb	Ishikawa Zone III	Palmar digital artery	Proximal nail bed vein	Vein	Not measured	0.6	3	14
6	Right thumb	Ishikawa Zone III	Palmar digital artery	Proximal nail bed vein	Vein	Not measured	1	4	14
7	Left index finger	Ishikawa Zone III	Palmar digital artery	Proximal nail bed vein	Vein	Not measured	0.6	6	13
8	Right thumb	Ishikawa Zone III	Palmar digital artery	Proximal nail bed vein	Vein	Not measured	0.6	12	11
9	Left ring finger	Ishikawa Zone III	Palmar digital artery	Proximal nail bed vein	Vein	Not measured	0.8	6	13
10	Left index finger	Dorsal composite block	Proximal subungual arch artery	Subcutaneous vein	Artery	0.3	Not measured	43	11
11	Right thumb	Dorsal composite block	Proximal subungual arch artery	Subcutaneous vein	Artery	0.3	Not measured	17	11
12	Right middle finger	Ishikawa Zone II	Proximal subungual arch artery	Subcutaneous vein	Artery	0.2	Not measured	3	15

### Surgical method

**Figure 1:**
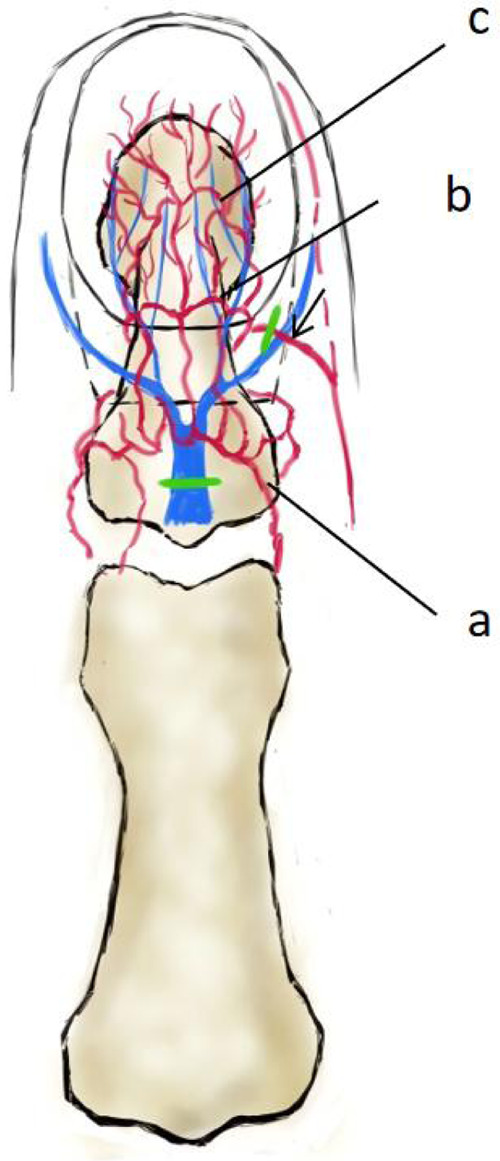
Schematic illustration of arterial reconstruction using the proximal subungual arch artery **(a)** nail fold arcade artery. **(b)** proximal subungual arch artery. **(c)** distal subungual arch artery. Red indicates artery, blue indicates vein, green indicates anastomosis site, and the dashed line indicates the transposed vessel..

**Figure 2:**
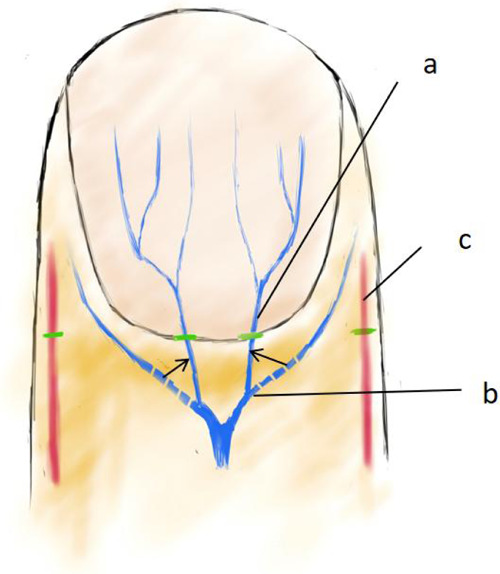
Schematic illustration of venous reconstruction using the proximal nail bed vein. **(a)** nail bed vein. **(b)** subcutaneous vein. **(c)** palmar digital artery. Red indicates artery, blue indicates vein, green indicates anastomosis, and the dashed line indicates the proximally transposed vessel.

All surgeries were performed under brachial plexus block anesthesia, with a pneumatic tourniquet applied to the proximal upper arm. Debridement of the Amputation Site: Initial debridement was carried out under direct vision to remove contaminated and non-viable tissue, with appropriate shortening of the bone ends. Subsequently, layer-by-layer debridement was performed under a microscope to identify the severed ends of arteries, nerves, and veins within the amputated segment. When the amputated tissue lacks a palmar digital artery, the proximal subungual arch artery stump and the nail bed nerve stump are identified beneath the interosseous ligament on both sides of the proximal nail bed of the amputated tissue to replace the distal palmar digital artery and nerve. On the proximal stump, the palmar digital artery is identified and appropriately dissected distally to beyond the digital arterial arch, where the artery diameter is similar to that of the proximal subungual arch artery. The distal end of the artery is then cut and ligated. When the amputated tissue lacks a subcutaneous vein, the proximal nail bed vein is identified at the amputated tissue stump at the nail root to replace the subcutaneous vein. The orifice of the nail bed vein may be cut obliquely or appropriately dilated to match the diameter of the proximal subcutaneous vein. On the proximal stump, the dorsal subcutaneous vein stump is identified. Repair of Bony, Articular, and Tendinous Structures: After aligning the bone ends, fixation was achieved using Kirschner wires. If the joint at the amputation site was destroyed, arthrodesis was performed. Injured tendons, joint capsules, and ligaments were repaired using non-absorbable sutures. Vascular and neural anastomosis: The artery, nerve, and vein at the stump were anastomosed sequentially. Arterial supply and venous drainage were successfully reconstructed in all 12 cases in this group. Among them, three cases of amputated tissue lacked a palmar digital artery for anastomosis, and the arterial anastomosis was modified by using the proximal subungual arch artery within the amputated tissue to replace the palmar digital artery for restoring arterial supply. Under a microscope with 10  ×   magnification, the proximal subungual arc artery stump of the transected tissue was anastomosed end-to-end with the proximal palmar digital artery using the two-point method, and the subungual nerve was anastomosed to the palmar digital nerve using the epineural suture technique. the proximal end of the dorsal subcutaneous vein was anastomosed end-to-end using the two-point method (Figure 1), with an artery-to-vein anastomosis ratio of artery 1: vein 1-2; Among them, nine cases of amputated tissue lacked a subcutaneous vein for anastomosis, and the venous anastomosis was modified by using the proximal nail bed vein within the amputated tissue to replace the subcutaneous vein for restoring venous drainage. Under a microscope, the palmar digital artery was anastomosed end-to-end using the two-point fixation method, and the palmar digital nerve stumps were coapted using the epineural suture technique. Subsequently, the proximal nail bed vein, serving as a substitute for the subcutaneous vein, was anastomosed end-to-end with the subcutaneous vein stump at the nail root using the two-point fixation method (Figure 2). The arteriovenous anastomosis ratio was artery 2: vein 1-2. Finally, the edges of the wound were closed with 5-0 absorbable sutures, covered with sterile dressings, and the affected hand was immobilized in a resting position using a medical polymer splint.

In cases where the palmar digital artery was unavailable, arterial inflow was reconstructed by end-to-end anastomosis between the proximal subungual arch artery of the amputated segment and the proximal stump of the palmar digital artery. Venous outflow was simultaneously restored by anastomosing the dorsal subcutaneous vein. Note: a, nail fold arcade artery; b, proximal subungual arch artery; c, distal subungual arch artery. Red indicates artery, blue indicates vein, green indicates anastomosis site, and the dashed line indicates the transposed vessel.

In cases where no suitable subcutaneous vein was available, venous outflow was reconstructed by anastomosing the proximal nail bed vein of the amputated segment to the dorsal subcutaneous vein of the proximal stump.Arterial inflow was concurrently established via standard palmar digital artery anastomosis. Note: a, nail bed vein; b, subcutaneous vein; c, palmar digital artery. Red indicates artery, blue indicates vein, green indicates anastomosis, and the dashed line indicates the proximally transposed vessel.

## Observation indicators

Intraoperative measurement of the diameter of the proximal subungual arch artery used as an alternative to the palmar digital artery, or of the proximal nail bed vein used as an alternative to the subcutaneous vein, and record the duration of ischemia and the duration of the procedure; postoperatively, monitor the survival of the replanted tissue and assess for complications such as vascular crisis, wound infection, nonunion of fractures, and tissue necrosis; Finally, during follow-up, assess the function of the affected finger using the Fingertip Injuries Outcome Score (FIOS). Scoring is based on ten criteria: nail shape, finger length, fullness, fracture healing, aesthetics, sensation, pain, range of motion, grip strength, and return to work. The total score is 32 points; a score of 12 or less is considered excellent, 13–18 points is considered good, 19–24 points is fair, and scores greater than 24 are classified as (7).

## Postoperative management

Postoperatively, routine symptomatic treatments were administered, including infection prevention, anticoagulation therapy, and antivasospastic therapy. Conventional postoperative care for digital replantation was provided. The external fixation was removed at 3 weeks postoperatively, and gradual active and passive functional training was initiated. Radiographs were taken at 2 to 3 months postoperatively to assess fracture healing, after which the Kirschner wires were removed. The wound was kept dry and clean until Kirschner wire removal.

## Results

In the 3 cases (3 fingers) where the proximal subungual arch artery as an alternative to the palmar digital artery, the arterial diameters were 0.2 mm, 0.3 mm, and 0.3 mm, with a mean diameter of 0.27 mm. In the 9 cases (9 fingers) where the proximal nail bed veins s an alternative to the subcutaneous vein, the venous diameters ranged from 0.6 mm to 1.0 mm, with a mean diameter of 0.72 mm. The warm ischemia time for the 12 amputated digits ranged from 3 h to 11 h, with a mean of 4.75 h. The duration of replantation surgery for the 12 cases ranged from 0.5 h to 2.83 h, with a mean of 1.97 h. All 12 replanted digits in the 12 cases survived successfully, with no occurrence of vascular crisis, wound infection, bony nonunion, or tissue necrosis. The follow-up period ranged from 3 to 43 months, with a mean of 12.17 months. The function scores of the affected fingers ranged from 11 to 15 points, with a mean of 12.5 points. The outcomes were excellent in 6 cases and good in 6 cases. (Table 1).

## Case presentation: case 1

**Figure 3:**
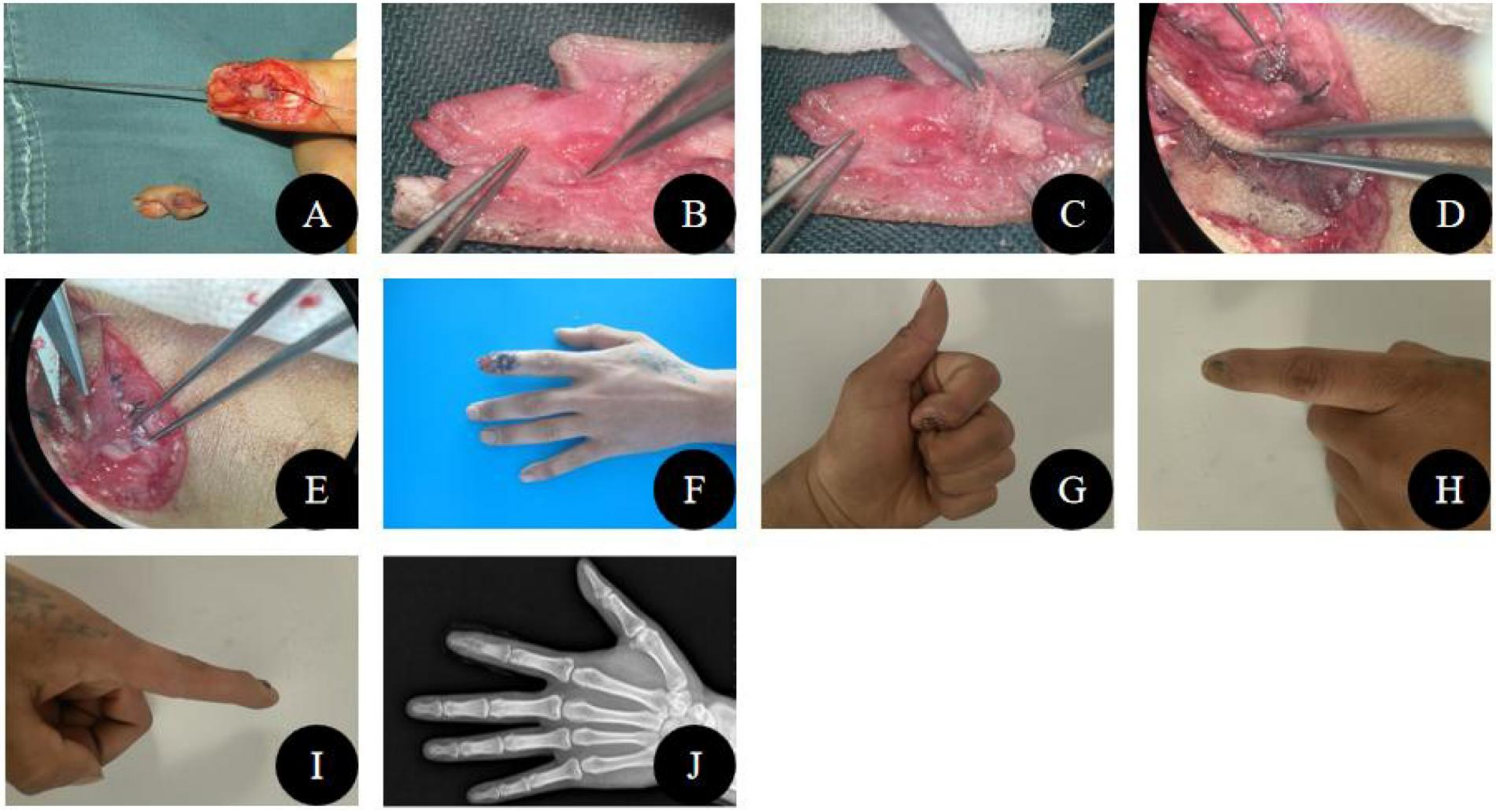
Case of arterial reconstruction using the proximal subungual arch artery as an alternative to the palmar digital artery. **(A)** Amputation of the tissue flap on the dorsal side of the left index finger's distal phalanx. **(B)** The proximal subungual artery on the radial side was identified during surgery. **(C)** The dorsal subcutaneous vein was identified during surgery. **(D)** Intraoperative view showing anastomosis of the proximal subungual arch artery and the nail bed nerve on the radial side to the palmar digital artery and nerve, respectively. **(E)** One dorsal subcutaneous vein was anastomosed during surgery. **(F)** Survival of the replanted tissue. **(G–I)** Nail growth was observed during the 16-month postoperative follow-up. **(J)** An x-ray taken two months after surgery showed that the fracture of the left index finger had healed well, and the internal fixation was removed.

A 30-year-old male patient presented with an amputation of the dorsal composite tissue block of the distal phalanx of the left index finger caused by an electric fan blade injury four hours prior to admission. Upon admission, physical examination revealed complete amputation of the dorsal tissue block of the distal phalanx of the left index finger. The insertion of the extensor tendon at the distal phalanx was located within the amputated tissue. Most of the distal phalanx, the palmar digital artery, and the nerve remained on the proximal stump, and no palmar digital artery was available for anastomosis within the amputated tissue. Emergency replantation of the amputated tissue block of the distal phalanx of the left index finger was performed. During the procedure, the arterial anastomosis was modified by using the proximal subungual arch artery within the amputated tissue to replace the palmar digital artery and anastomosing it with the proximal palmar digital artery to restore arterial supply. Simultaneously, standard anastomosis of the proximal dorsal subcutaneous vein was performed, and the insertion of the extensor tendon was reconstructed. The insertion of the extensor tendon was reconstructed. The total ischemia time for the amputated part was 7 h, and the operation duration was 2 h. The replanted tissue survived until discharge, with no complications such as vascular crisis, wound infection, fracture nonunion, or tissue necrosis observed. At the 43-month postoperative follow-up, the grafted finger appeared well-formed with no obvious atrophy; nail growth was good; flexion and extension at the distal interphalangeal joint were good; the affected finger received a functional score of 11; the outcome was excellent (Figure 3).

## Case 2

**Figure 4:**
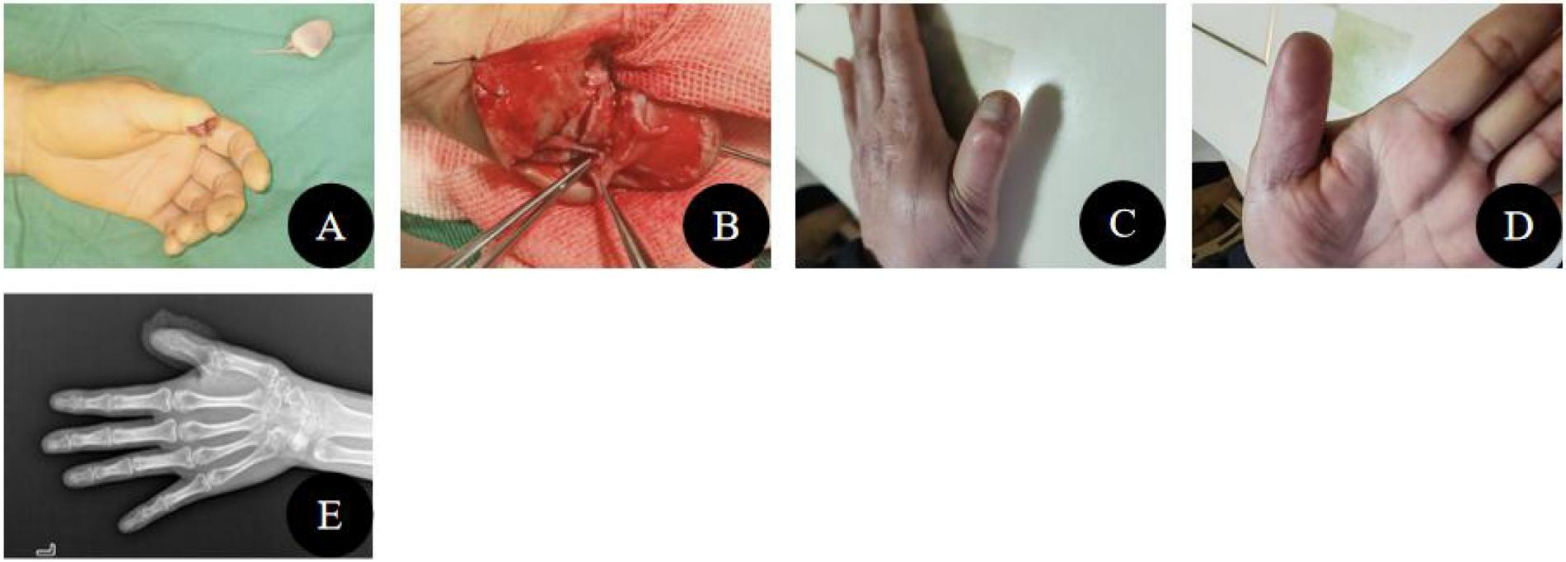
Case of venous reconstruction with proximal nail bed vein replacing the subcutaneous vein. **(A)** Amputation of the left thumb at the level of the nail root. **(B)** Anastomosis of a proximal nail bed vein with a dorsal subcutaneous vein. **(C)** Follow-up at 19 months postoperatively showing smooth nail growth. **(D)** Follow-up at 19 months postoperatively showing a well-preserved contour. **(E)** An x-ray taken two months after surgery showed that the fracture of the left thumb had healed well, and the internal fixation was removed.

A 56-year-old female patient presented with distal phalanx amputation of the left thumb caused by a machine twisting injury one hour prior to admission. Emergency replantation of the distal phalanx of the left thumb was performed upon admission. No subcutaneous vein suitable for anastomosis was found on the volar side of the amputated tissue. During the procedure, the venous anastomosis was modified by using the proximal nail bed vein within the amputated tissue as an alternative to the subcutaneous vein and anastomosing it with the proximal subcutaneous vein to restore venous drainage. Simultaneously, standard anastomosis of the palmar digital artery was performed to restore arterial supply. Postoperatively, the replanted tissue survived until discharge without complications such as vascular crisis, wound infection, fracture nonunion, or tissue necrosis. At the 19-month postoperative follow-up, the grafted finger appeared plump with no obvious atrophy, and the nail was growing well. The affected finger received a functional score of 11, indicating an excellent outcome. (Figure 4).

## Discussion

Distal fingertip amputation is a common clinical injury. Since the proximal phalanx and joint remain intact, replantation preserves most of the joint function of the affected digit, and successful replantation can maximize the restoration of finger function and aesthetics. Yano et al. considered distal fingertip replantation to be one of the best indications for microsurgery (5, 6).

Dong et al. identified multiple factors influencing the survival of replanted digits, including age, smoking history, hypertension, diabetes mellitus, amputation level, mechanism of injury, number of amputated digits, preservation method of the amputated digit, presence of concomitant injuries, ischemia time, operative time, surgeon experience, postoperative care, and blood circulation reconstruction (8–20). Among these, the reconstruction of blood circulation in the amputated distal fingertip tissue is the key factor for replantation survival, which includes both arterial inflow and venous outflow reconstruction (6).

Distal fingertip amputations can be generally classified into Ishikawa zones I, II, III, and IV, as well as special types such as palmar and dorsal fingertip tissue amputations. For Ishikawa zones I and II amputations, the palmar digital artery at the amputation stump is small in diameter with low pressure, and the subcutaneous vein is too tiny for reliable anastomosis. Murat et al. demonstrated that for zone I and II amputations, anastomosis of the palmar digital artery alone, without venous anastomosis, is sufficient to maintain blood balance through external bleeding, resulting in a high survival rate (21, 22). For Ishikawa zones III and IV amputations, the palmar digital artery is larger in diameter, anatomically constant, and has higher pressure, making arterial reconstruction easier than in zones I and II. However, the higher arterial pressure necessitates venous outflow reconstruction, for which subcutaneous vein anastomosis is the first choice (23).

Clinically, some amputated digits have no available subcutaneous vein for anastomosis. Some reports have described unconventional methods to maintain blood balance, such as arterialization of the vein or reducing the arterial anastomotic diameter to lower arterial pressure, with certain clinical efficacy (24, 25). In our study, we found that in the absence of a subcutaneous vein, anastomosis of the proximal nail bed vein provides an alternative for venous outflow reconstruction. The nail bed vein has a relatively large diameter. In this study, 9 patients (9 digits) with no available subcutaneous vein underwent nail bed vein anastomosis to reconstruct venous return, resulting in minimal postoperative bleeding, survival of all digits, and high-quality outcomes.

Arterial and venous distribution of the nail bed: The arterial and venous anatomy of the nail bed has been described in previous studies (26). The arterial vascular plexus of the fingertip nail apparatus is dense and primarily originates from three transverse arcade arteries: the nail fold arcade artery, the proximal subungual arch artery, and the distal subungual arch artery (Figure 1). The proximal subungual arch artery is also known as the germinal matrix arcade, and the distal subungual arch artery is known as the sterile matrix arcade. These three transverse arches are interconnected by longitudinal vascular networks. The nail fold arch receives blood supply from the dorsal collateral artery of the middle phalanx and the dorsal branch of the palmar digital artery at the level of the distal interphalangeal joint. The collateral branches of the palmar digital artery at the distal phalanx pass through the interosseous ligament and give off proximal and distal branches. The proximal branch joins the dorsal branch of the palmar digital artery at the distal interphalangeal joint to form the proximal subungual arch artery, while the distal branch joins the pulp artery to form the distal subungual arch artery (27). Based on the homology between the hand and foot, there are numerous reports on using toe nail bed to repair finger nail bed defects, and the vascular anatomy of the toe nail bed has also been well documented (28–31).

In our study, the diameters of the proximal subungual arch artery in three cases were 0.2 mm, 0.3 mm, and 0.3 mm. With the advancement of microsurgical instruments and techniques, anastomosis of vessels as small as 0.2 mm is no longer a problem. Small veins of the nail bed gradually converge from distal to proximal to form a larger proximal nail bed vein. A relatively consistent pattern is that the combined veins on both sides of the nail bed converge at the nail root into a midline vein that drains into the dorsal subcutaneous tissue (Figure 2). In our 9 cases, the diameters of the nail bed veins ranged from 0.6 mm to 1.0 mm (mean: 0.72 mm), which are comparable to the diameters of the lateral subcutaneous vein and palmar subcutaneous vein, making anastomosis relatively easy.

For palmar fingertip tissue amputation, the amputation involves the palmar digital artery and palmar subcutaneous vein. Anastomosis of the palmar digital artery for arterial inflow and the palmar subcutaneous vein for venous outflow results in a high replantation success rate. For dorsal fingertip tissue amputation, since the palmar digital artery is located within the palmar tissue, the arterial supply to the dorsal amputated tissue originates from the nail fold arch, proximal subungual arch artery, and distal subungual arch artery. The nail fold arch and distal subungual arch artery are too small in diameter for reliable anastomosis; therefore, dorsal tissue replantation requires anastomosis of the proximal subungual arch artery. In three cases of distal fingertip amputation without a palmar digital artery available for anastomosis, we successfully identified the proximal subungual arch artery on the lateral side of the proximal nail bed, with diameters ranging from 0.2 to 0.3 mm. After adequate mobilization, the proximal palmar digital artery was anastomosed to the proximal subungual arch artery to reconstruct arterial inflow. All replanted tissues survived with high-quality outcomes.

Surgical considerations: ① Perform thorough debridement of the amputation stump to prevent infection. ② Identify and mark the cut ends of the artery, nerve, and vein under microscopic debridement. Carefully evaluate the arterial and venous status of the amputated tissue and select the appropriate method for blood circulation reconstruction. If no palmar digital artery is available for anastomosis, the proximal subungual arch artery should be identified for arterial reconstruction. If no subcutaneous vein is available for anastomosis, the proximal nail bed vein should be identified for venous reconstruction. ③ For fracture fixation, the Kirschner wire should be inserted in a retrograde manner: first insert the wire from the distal bone stump, then after approximating the stumps, advance the wire to fix the fracture, avoiding repeated wire passes that may damage the vessels. ④ Perform thorough debridement of the vascular stumps. If a vascular defect exists, a free forearm subcutaneous vein graft may be used. For arterial grafting, the vein graft should be reversed. Perform high-quality vascular anastomosis to prevent vascular crisis and digit necrosis. ⑤ Perform nerve anastomosis to improve the functional quality of the replanted digit.

## Conclusion

The proximal subungual arch artery and the nail bed vein can be anastomosed under a microscope. Anastomosis of the proximal subungual arch artery can replace the palmar digital artery to restore arterial supply to the distal amputated tissue block, while anastomosis of the proximal nail bed vein can replace the subcutaneous vein to restore venous drainage of the distal amputated tissue block. For patients with distal fingertip amputation injuries lacking a palmar digital artery or subcutaneous vein available for anastomosis, replantation can be performed by intraoperatively anastomosing the proximal subungual arch artery as an alternative to the palmar digital artery or the proximal nail bed vein as an alternative to the subcutaneous vein to restore arterial supply or venous drainage. This approach effectively improves the replantation rate, survival rate, and survival quality of the replanted tissue, and is worthy of widespread clinical application.

## Data Availability

The original contributions presented in the study are included in the article/Supplementary Material, further inquiries can be directed to the corresponding author.
